# Rewiring the immune response in lung cancer: current progress in bispecific antibodies, CAR-T therapy, and the rise of *in vivo* CAR-T platforms

**DOI:** 10.3389/fimmu.2026.1772428

**Published:** 2026-04-21

**Authors:** Lei He, Yi Sun, Bowen Ma, Guojun Lang, Jianguo Wen, George R. Blumenschein, Hong Ma

**Affiliations:** 1 NANOIMMUNOCEL Inc, Houston, TX, United States; 2 Sanyou Biopharmaceuticals Co., Ltd., Cambridge, MA, United States; 3 School of Natural Sciences, Rice University, Houston, TX, United States; 4 University of Texas Health Science Center at Houston (UT) Health, School of Biomedical Informatics, Houston, TX, United States; 5 The University of Texas MD Anderson Cancer Center, Department of Thoracic/Head and Neck Medical Oncology, Houston, TX, United States

**Keywords:** bispecific antibody, CAR T-cell therapy, *in vivo* CAR programming, lung cancer, mRNA, non–small cell lung cancer, small cell lung cancer

## Abstract

Lung cancer remains the leading cause of cancer mortality worldwide and continues to impose a major clinical burden, particularly in advanced non–small cell lung cancer (NSCLC) and small-cell lung cancer (SCLC). Although targeted therapies, antiangiogenic agents, immune checkpoint inhibitors, and antibody-drug conjugates have improved outcomes in selected patients, durable responses remain limited by primary and acquired resistance. Here, we comprehensively review recent progress in immunologically oriented therapeutic strategies for lung cancer, focusing on bispecific antibodies, chimeric antigen receptor (CAR) T-cell therapy, and emerging *in vivo* CAR-engineering modalities. We further elaborate on the clinical rationale, latest translational and early clinical evidence, and key challenges, including on-target, off-tumor toxicity, cytokine release syndrome, limited T-cell persistence, insufficient tumor trafficking, and immunosuppression within the tumor microenvironment. Taken together, we find that while bispecific antibodies currently show favorable efficacy and safety in lung cancer; advances in CAR design and *in vivo* delivery may broaden the applicability of CAR-T therapy in this setting.

## 1 Introduction

**Figure 1:**
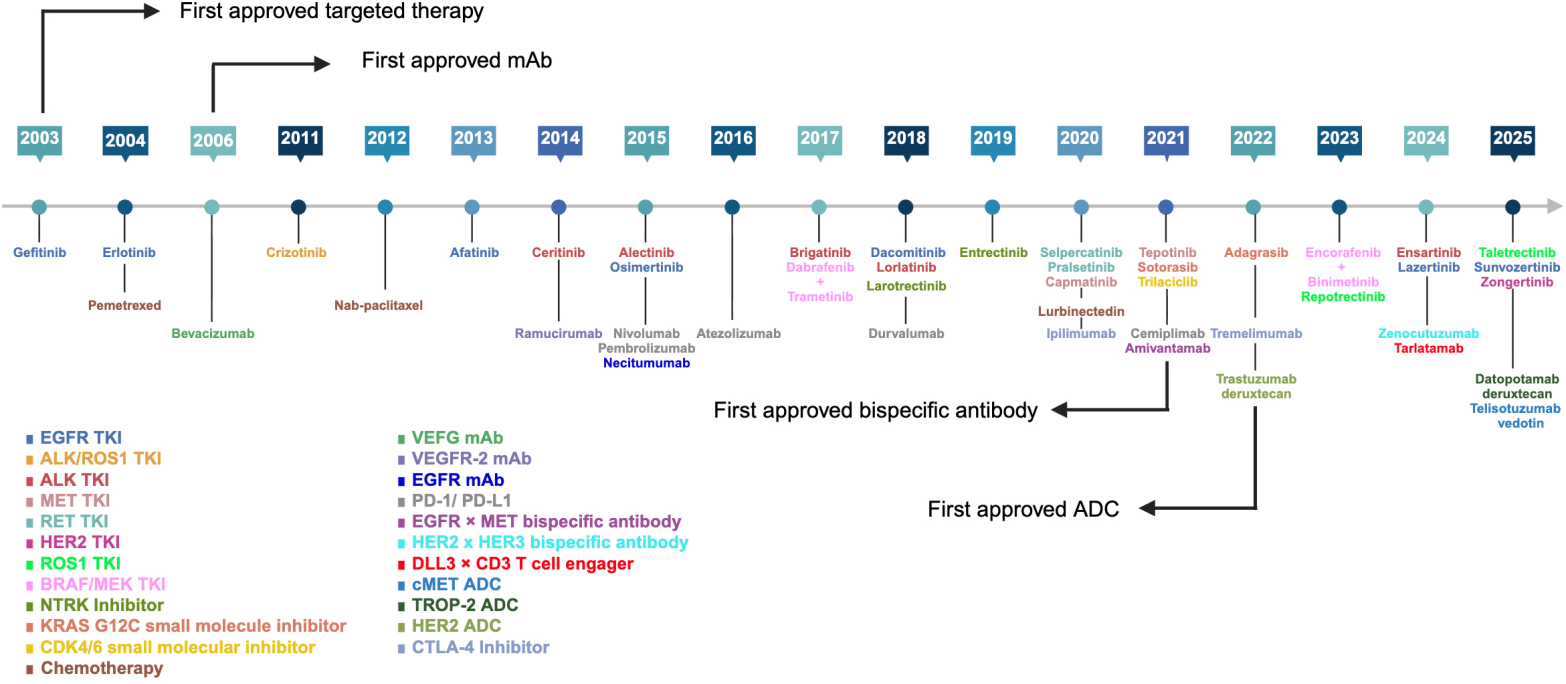
FDA-approved therapies for lung cancer (2000-October 2025). From 2000 to October 2025, 46 agents received FDA approval for lung cancer, including 22 TKIs, 7 immune checkpoint inhibitors, 3 chemotherapies, 3 mAbs, 3 bispecific antibodies, 3 ADCs, 3 small-molecule inhibitors, and 2 NTRK inhibitors. The figure does not include 11 chemotherapy agents approved before 2000: vincristine, cyclophosphamide, doxorubicin, cisplatin, etoposide, carboplatin, vinorelbine, gemcitabine, paclitaxel, topotecan, and docetaxel. ADC, antibody drug conjugate; ALK, anaplastic lymphoma kinase; BRAF, B-Raf proto-oncogene, serine/threonine kinase; CDK4/6, cyclin-dependent kinases 4 and 6; CTLA-4, cytotoxic T-lymphocyte-associated protein 4; c-MET, c-mesenchymal-epithelial transition factor; DLL3, delta-like ligand 3; EGFR, epidermal growth factor receptor; HER2, human epidermal growth factor receptor 2; HER3, human epidermal growth factor receptor 3; KRAS, KRAS proto-oncogene, GTPase; mAb, monoclonal antibody; MEK, mitogen-activated protein kinase; NTRK, neurotrophic tyrosine receptor kinase; PD-1, programmed cell death protein-1; PD-L1, programmed death-ligand 1; RET, rearranged during transfection; ROS1, c-ros oncogene 1; TKI, tyrosine kinase inhibitor; TROP-2, trophoblast cell-surface antigen 2; VEGF, vascular endothelial growth factor; and VEGFR-2, vascular endothelial growth factor receptor-2. Created with BioRender.com.

Lung cancer continues to be the leading cause of cancer mortality globally, with approximately 2.5 million new diagnoses and 1.8 million deaths annually (1). Non-small cell lung cancer (NSCLC) accounts for ~85% of cases, while small-cell lung cancer (SCLC) represents ~15% (2–4). Despite advances in early detection, >50% of NSCLC and ~70% of SCLC patients present with metastatic disease, with 5-year survival remaining at ~25% in NSCLC and 2-year survival remaining at 15% in SCLC (2, 4–7). The advent of targeted therapies (e.g., EGFR/ALK/ROS1/MET inhibitors), antiangiogenic monoclonal antibodies (VEGF inhibitors), and immune checkpoint inhibitors (PD-1/PD-L1 blockade) has revolutionized outcomes in NSCLC (**Figure 1**); however, primary/acquired resistance limits durable responses (8). In SCLC, first-line platinum-etoposide and anti-PD-L1 (atezolizumab/durvalumab) improved median overall survival (OS) to approximately 12–13 months, though most patients relapse within 6 months (9, 10). Newer agents, including the DNA minor groove binder, lurbinectedin, and the DLL3×CD3 bispecific antibody tarlatamab, have recently been approved, but therapeutic options in this disease remain limited (11, 12). These substantial unmet needs in both NSCLC and SCLC underscore the urgent need for novel therapeutic strategies, with bispecific antibodies and next-generation cellular therapies offering promising avenues to improve patient outcomes.

## 2 Bispecific antibodies

Bispecific antibodies have emerged as a transformative class of therapeutics, capable of simultaneously engaging multiple pathways to advance treatment paradigms in lung cancer. As of October 2025, three bispecific agents have received regulatory approval, including amivantamab and zenocutuzumab for NSCLC, and tarlatamab for SCLC.

Amivantamab, an EGFR×MET bispecific antibody, is approved for EGFR-mutant NSCLC. In first-line EGFR-mutated NSCLC, the phase III study (NCT04487080) demonstrated that an amivantamab-based regimen significantly improved median progression-free survival (PFS) compared with standard osimertinib (23.7 vs 16.6 months; hazard ratio [HR] 0.70; 95% CI, 0.58 to 0.85; *P* < 0.001) *(*13). For first-line EGFR-mutated NSCLC with exon 20 insertion mutation, the phase III trial (NCT04538664) demonstrated a median PFS of 11.4 months with amivantamab plus chemotherapy, compared with 6.7 months with chemotherapy alone (HR, 0.40; 95% CI, 0.30 to 0.53; *P* < 0.001) (14). For second-line EGFR-mutated NSCLC, the phase III study (NCT04988295) showed a median PFS of 6.3 months with amivantamab plus chemotherapy compared with 4.2 months with chemotherapy alone (HR, 0.48; 95% CI, 0.36 to 0.64; *P* < 0.001) (15). Additionally, two other EGFR×MET bispecifics, EMB-01 and MCLA-129, are in phase I/II clinical trials (Appendix 1).

Zenocutuzumab, a HER2/HER3 bispecific antibody, was approved for advanced NRG1 fusion-positive NSCLC and pancreatic cancer following prior therapy (16). The registrational phase II study (NCT02912949) reported an objective response rate (ORR) of 29% (95% CI, 25 to 59) and a median duration of response (DoR) of 12.7 months (95% CI, 20 to 39) in *NRG1+* NSCLC patients (17).

In SCLC, tarlatamab, a delta-like ligand 3 (DLL3)–targeting bispecific T-cell engager (TCE), was approved for previously treated extensive-stage SCLC (18). Its pivotal phase II trial yielded an ORR of 40% (95% CI, 29 to 52), and the median PFS was 4.9 months (95% CI, 2.9 to 6.7) (12). Additionally, two other DLL3 × CD3 TCEs, BI764532 and PN328/MK6070 are under phase I/II trials (Appendix 1).

### 2.1 Emerging PD-1/PD-L1×VEGF

Clinical evidence demonstrated that VEGF not only promotes angiogenesis but also contributes to immune evasion by enhancing the suppressive activity of regulatory T cells and tumor-associated macrophages, inhibiting dendritic cell maturation, and restricting effector T-cell infiltration into tumors (19). Inhibition of VEGF can normalize tumor vasculature and enhance immune cell infiltration, thereby enhancing the effects of PD-1/PD-L1 blockade (20, 21). This hypothesis was validated in the phase III trial, where adding atezolizumab to bevacizumab plus chemotherapy significantly improved OS (19.2 vs 14.7 months; HR, 0.78; 95% CI, 0.64 to 0.96; *P* = 0.02) compared with bevacizumab plus chemotherapy (22).

Building on this foundation, the therapeutic landscape now features a rapidly expanding pipeline of PD-1/PD-L1×VEGF bispecific and multispecific agents in both early- and late-phase development. In China, ivonescimab (AK112) is the first approved PD-1 × VEGF bispecific antibody for EGFR-mutated nonsquamous NSCLC after progression on tyrosine kinase inhibitor (TKI) therapy and for first-line treatment of PD-L1–positive NSCLC (23). In a phase III trial (NCT05184712), ivonescimab plus chemotherapy significantly improved median PFS versus placebo plus chemotherapy (7.1 vs 4.8 months; HR, 0.46; 95% CI, 0.34–0.62; P < 0.001) in patients with EGFR-TKI–refractory NSCLC in China (24). The corresponding phase III global trial (NCT05899608) in patients with NSCLC who had progressed on EGFR-TKI treatment enrolled 438 patients. At the primary analysis, median PFS was 6.8 months with ivonescimab plus chemotherapy versus 4.4 months with placebo plus chemotherapy (HR, 0.52; 95% CI, 0.41-0.66; *P* < 0.0001). The median OS was 16.8 months in the ivonescimab group and 14.0 months in the placebo group, with a positive trend (HR, 0.79; 95% CI, 0.61 to 1.01; *P* = 0.057) *(*25).

Additionally, ivonescimab was evaluated in the phase III trial (NCT05499390) as first-line therapy for patients with PD-L1–positive NSCLC, compared with pembrolizumab. Ivonescimab significantly improved median PFS (11.1 vs 5.8 months; HR, 0.51; 95% CI, 0.38–0.69; *P* <.0001) *(*26). In another phase III trial (NCT05840016), ivonescimab plus chemotherapy was evaluated as first-line treatment for NSCLC, regardless of PD-1 status, compared with tislelizumab plus chemotherapy. Ivonescimab prolonged median PFS (11.1 vs 6.9 months; HR, 0.60; 95% CI, 0.46–0.78; *P* <.0001) *(*27, 28). In addition, ivonescimab was also evaluated in a phase II basket NSCLC study (NCT04736823), demonstrating ORRs ranging from 40% to 68.4% (**Table 1**) (29). Among all these studies, ivonescimab demonstrated a manageable safety profile. The most common grade ≥ 3 adverse events were decreased blood counts. Immune-related AEs (irAEs) included rash, dermatitis, and interstitial lung disease. Adverse events related to VEGF included proteinuria, bleeding, and hypertension (**Table 2**) (24–29).

**Table 1:** Efficacy outcomes of PD-1/PD-L1 and VEGF-targeting bispecific antibodies in lung cancer clinical trials.

Products/NCT/phase	N	Diagnosis	Interventions	Efficacy results
PD-1 × VEGF (Ivonescimab/AK112) (24) NCT05184712 Phase III	321 Ivonescimab group (n = 161) Placebo (n = 161)	Locally advanced or metastatic (stage IIIB, IIIC, or IV) NSCLC harboring EGFR mutations who experienced disease progression following prior EGFR-TKI therapy.	Ivonescimab plus pemetrexed and carboplatin Q3W for four cycles followed by Ivonescimab plus pemetrexed maintenance treatment *vs* placebo plus pemetrexed and carboplatin Q3W for four cycles followed by placebo plus pemetrexed maintenance treatment	Median follow-up: 7.89 months Ivo vs Placebo Median PFS per IRCC (month):7.1 vs 4.8 (HR, 0.46; 95% CI, 0.34-0.62; P < 0.001) ITT population (Ivo vs Placebo, 160 vs 161) ORR: 50.6% (95% CI, 42.6% to 58.6%) vs 35.4% (95% CI, 28.0% to 43.3%) DCR: 93.1% (95% CI, 88% to 96.5%) vs 83.2% (95% CI, 76.5% to 88.6%]) Median DoR (month): 6.6 (95% CI, 4.3 to 7.6) vs 4.2 (95% CI, 3.0 to 4.7)
PD-1 × VEGF (Ivonescimab/AK112) (26) NCT05499390 Phase III	198 (Ivonescimab) vs 200 (Pembrolizumab)	1st line therapy for locally advanced or metastatic, PD-L1 positive NSCLC	Ivonescimab Q3W *vs* pembrolizumab Q3W	Median follow-up: 8.7m Ivo vs Pembro Time to response (month): 1.5 vs 2.5 Median PFS (month):11.1(95% CI, 7.3 to not estimable) vs 5.8 (95% CI 5.0-8.2); HR, 0.51 (95% CI, 0.38-0.69; P <0.0001) ORR:50% (95% CI 43-57) vs 39% (95% CI 32-46) DCR (Ivo vs Pembro): 90% (95% CI 43-57) vs 71% (95%CI 64-77) Median DoR (month): Not reached in both arms
PD-1 × VEGF (Ivonescimab/AK112) (27, 28) NCT05840016 Phase III	266 (Ivonescimab) vs 266 (tislelizumab)	1^st^ line therapy for advanced squamous NSCLC	Ivonescimab plus paclitaxel and carboplatin for up to four cycles, followed by ivonescimab maintenance vs. tislelizumab plus paclitaxel and carboplatin for up to four cycles, followed by tislelizumab maintenance	Median follow-up: 10.4 month Ivo vs Tislezumab Median PFS (month):11.1(95% CI, 9.9 to NE) vs 6.9 (95% CI, 5.8 to 8.6); HR,0.60 (95% CI, 0.46 to 0.78; P <0.0001) ORR: 76% (202/266; 95% CI, 70 to 81) vs 67%(177/266; 95% CI, 61 to 72) Median DoR (month): 11.2 (95% CI 8.5 to NE) vs 8.4 (95% CI 5.7 to NE)
PD-1 × VEGF (Ivonescimab/AK112) (29) NCT04736823 Phase II	82 (total efficacy evaluable pt) Cohort 1: n=43 Cohort 2: n=19 Cohort 3: n=20	Advanced NSCLC Cohort 1: Previously untreated advanced NSCLC without EGFR or ALK mutation Cohort 2: Advanced NSCLC with EGFR-sensitive mutations Cohort 3: Advanced NSCLC who failed systemic platinum-based chemotherapy and PD-1/PD-L1	Ivonescimab (AK112) at 10 mg/kg or 20 mg/kg administered every three weeks (Q3W) in combination with chemotherapy for four cycles, followed by maintenance therapy with either Ivonescimab monotherapy or Ivonescimab plus chemotherapy	Cohort 1: ORR: 53.5% (23/43; 95% CI, 36.9 to 67.1) DCR: 93.0% (40/43; 95% CI, 80.9 to 98.5) Median DoR(month): NR Cohort 2: ORR: 68.4% (13/19; 95% CI, 43.4 to 87.4) DCR: 94.7%(18/19; 95%CI, 74.0 to 99.9) Median DoR(month): 8.38 (95% CI, 5.5 to NE) Cohort 3 ORR: 40%(8/20; 95% CI, 19.1 to 63.9) DCR: 70%(14/20; 95%CI, 45.7 to 88.1) Median DoR(month): 7.5 (95%CI, 2.3 to NE)
PD-1 × VEGF (SSGJ-707) (30) NCT06361927 Phase II	76	Treatment-naive advanced NSCLC	Single-arm study; SSGJ-707 at 5mg/kg Q3W, 10 mg/kg Q3W, 20 mg/kg Q3W and 30 mg/kg Q3W	5mg/kg Q3W (n=31) ORR: 29.6% (8/27) DCR: 85.2% (23/27) 10mg/kg Q3W (n=34) ORR: 61.8%(21/34) DCR: 97.1% (33/34) 20mg/kg Q3W (n=12) ORR: 54.5% (6/11) DCR: 90.9% (10/11) 30mg/kg Q3W (n=6) ORR: 25% (1/4) DCR: 90.9% 75% (3/4)
PD-1 × VEGF (HB0025) (31) NCT04678908 Phase I	30	Advanced solid tumor	Dose escalation study; HB0025 was administered at 0.01, 0.03, 0.1, 0.3, 1.0, 3.0, 6.0, 10, 12, 20, and 30 mg/kg Q2W.	Among 22 evaluable pts dosed at ≥ 3 mg/kg Q2W: ORR: 9.1% (2/22, including 1 colorectal cancer pt achieved CR and 1 NSCLC pt achieved PR) DCR: 50% (11/22)
PD-L1 × VEGF (Pumitamig/BNT327) (32) NCT05879068 Phase II	70	2L SCLC who progressed on chemo with or without PD-(L)1 treatment	Pumitamig 30 mg/kg Q3W + Paclitaxel 175 mg/m2 Q3W for 5 cycles, followed by Pumitamig maintenance therapy	Median follow-up(month):17.9 ORR: 41.5% (29/70; 95% CI, 29.4 to 54.4) DCR: 87.7% (61/70; 95% CI, 77.2 to 94.5) Median PFS (month): 5.5 (95%CI, 4.1 to 7.2) Median OS (month): 14.3 (95% CI, 10.9 to 19.9)
PD-L1 × VEGF (Pumitamig/BNT327) (33) NCT05844150 Phase II	50 (48 for efficacy analysis)	1st line extensive-stage small-cell lung cancer (ES-SCLC)	Pumitamig 30 mg/kg +etoposide + platinum Q3W for 4 cycles followed by pumitamig maintenance therapy	Overall ORR: 87.5% (42/48) Confirmed ORR: 85.4% (41/48) DCR:100% Median DOR (month):5.5 (95% CI, 3.8 to 6.8) Median PFS (month):6.9 (95% CI, 4.3 to 8.2) Median OS (month): not matured yet
PD-L1 × VEGF (IMM2510) (35) NCT05972460 Phase I	33	Advanced solid tumor	Dose escalation study; IMM2510 was administered at 0.007, 0.03, 0.1, 0.3, 1.0, 3.0, 6.0, 10, and 20mg/kg Q2W.	Among 25 response evaluable pts: 3 PR (including 2 NSCLC and 1 thymic carcinoma) 7 SD

CR, Complete response; DCR, Disease control rate; DOR, Duration of response; IRCC, Independent radiographic review committee; NCT, National Clinical Trial number; NE, not estimable; NSCLC, Non-small cell lung cancer; ORR, Objective response rate; PFS, Progression-free survival; PR, Partial response; pt, Patient; SCLC, Small cell lung cancer; SD, Stable disease; 2L, Second line.

**Table 2:** Safety outcomes of PD-1/PD-L1 and VEGF-targeting bispecific antibodies in lung cancer clinical trials.

Products/NCT/phase	N	AESI related to VEGF	Most common AE
PD-1 × VEGF (Ivonescimab/AK112) (24) NCT05184712 Phase III	Ivonescimab + chemo group (n = 161) Placebo + chemo (n = 161)	*Ivo vs Placebo Proteinuria: 17.4% vs 8.1%* *Bleeding: 6.8% vs 5.0%* *Hypertension: 8.1% vs 3.1%*	Ivo vs Placebo Any grade TEAE: 99.4% (160/161) vs 97.5% (157/161) G3 or higher TEAE: 61.5% (99/161) vs 49.1% (79/161) White blood cell count decreased: 65.8% (106/161) vs 64.6% (104/161) Anemia: 63.4% (102/161) vs 73.3% (118/161) Neutrophil count decreased: 60.2% (97/161) vs 58.4% (94/161) Platelet count decreased: 48.4% (78/161) vs 41.0% (66/161) Aspartate aminotransferase increased: 44.1% (71/161) vs 31.1% (50/161) Alanine aminotransferase increased: 37.3% (60/161) vs 36.6% (59/161) Vomiting: 34.2% (55/161) vs 35.4% (57/161) Decreased appetite: 32.9% (53/161) vs 21.7% (35/161) COVID-19 infection: 30.4% (49/161) vs 20.5% (33/161) Constipation: 28.6% (46/161) vs 19.9% (32/161) Fatigue: 22.4% (36/161) vs 17.4% (28/161) γ-Glutamyl transferase increased: 21.7% (35/161) vs 13.7% (22/161) Proteinuria: 21.7% (35/161) vs 13.0% (21/161) Hypoalbuminemia: 20.5% (33/161) vs 14.3% (23/161) Hypercholesterolemia: 20.5% (33/161) vs 12.4% (20/161)
PD-1 × VEGF (Ivonescimab/AK112) (26) NCT05499390 Phase III	197 (Ivonescimab) vs 199 (Pembrolizumab)	*Ivo vs Pembro* *Any: 48% (94/197) vs 21% (42/199)* *Proteinuria: 31% (62/197) vs 10% (20/199)* *Hypertension: 16% (31/197) vs 3% (5/199)* *Hemorrhage: 15% (29/197) vs 11%(22/199)* *Arterial thromboembolism: 1%(2/197) vs 1% (1/199) Venous thromboembolism: 0 vs 1%(1/199)*	*Ivo vs Pembro* *Any grade TRAE: 90% (177/197) vs 82% (163/199)* *G3 or higher TRAE: 29% (58/197) vs 16% (31/99)* *Proteinuria 32% (62/197) vs 10% (20/199)* *AST increased 20% (39/197) vs 16% (31/199)* *Hypercholesterolemia 16% (32/197) vs 10% (20/199)* *Blood bilirubin increased 16% (31/197) vs 12% (23/199)* *Hypertension 16% (31/197) vs 3% (5/199)* *ALT increased 15% (29/197) vs 12% (24/199)* *Hypothyroidism 14% (28/197) vs 10% (19/199)* *Anemia 13% (26/197) vs 15% (29/199)* *Hypoalbuminemia 12% (23/197) vs 11% (22/199)* *Amylase increased 11% (22/197) vs 3% (6/199)* *Hyperglycemia 11% (22/197) vs 12% (23/199)* *Blood uric acid increased 11% (21/197) vs 8% (16/199)* *Hypertriglyceridemia 10% (20/197) vs 7% (14/199)* *Arrhythmia 10% (20/197) vs 11% (21/199)* *Rash 8% (15/197) vs 14%(28/199)*
PD-1 × VEGF (Ivonescimab/AK112) (27, 28) NCT05840016 Phase III	266 (Ivonescimab) vs 266 (tislelizumab)	*Ivo vs Tislelizumab Any: 46% (123/266) vs 23% (60/265)Proteinuria: 27% (72/266) vs 11% (29/265)Hemorrhage: 21% (57/266) vs 9% (25/265)Hypertension: 10% (27/266) vs 5% (12/265)Arterial thromboembolism: 1% (3/266) vs 0% Venous thromboembolism: 1% (2/266) vs 1% (3/265)Fistula: <1% (1/266) vs 0% (0/265)*	*Ivo vs Tislelizumab Any grade TRAE: 99% (264/266) vs 98% (261/265)G3 or higher TRAE: 64% (170/266) vs 54% (144/264)Alopecia 65% (174/266) vs 61% (162/265)Anemia 53% (141/266) vs 58% (153/265)Decreased neutrophil count 45% (120/266) vs 43% (113/265)Decreased white blood cell count 36% (96/266) vs 34% (90/265)Decreased platelet count 29% (76/266) vs 25% (66/265)Hypoesthesia 27% (71/266) vs 25% (65/265)Decreased appetite 22% (58/266) vs 24% (64/265)Increased alanine aminotransferase 20% (52/266) vs 20% (53/265)Pain in extremity 19% (50/266) vs 12% (33/265)Proteinuria 18% (48/266) vs 7% (19/265)Hypertriglyceridemia 17% (46/266) vs 12% (33/265)Hypoalbuminemia 16% (42/266) vs 10% (27/265)Increased aspartate aminotransferase 16% (42/266) vs 16% (42/265)Leukopenia 14% (38/266) vs 17% (45/265)Nausea 14% (38/266) vs 19% (51/265)Decreased weight 14% (37/266) vs 10% (27/265)Peripheral neuropathy 13% (34/266) vs 12% (32/265)Constipation 12% (33/266) vs 11% (30/265)Hyperuricemia 12% (33/266) vs 7% (18/265)Hypercholesterolemia 12% (32/266) vs 12% (32/265)Hemoptysis 12% (32/266) vs 4% (10/265)Increased blood urea 12% (32/266) vs 7% (19/265)Diarrhea 12% (31/266) vs 5% (12/265)Arthralgia 11% (30/266) vs 8% (20/265)Rash 11% (30/266) vs 11% (29/265)Increased γ-glutamyltransferase 11% (29/266) vs 12% (33/265)Hyperlipidemia 11% (29/266) vs 5% (12/265)Hypothyroidism 11% (28/266) vs 11% (29/265)Albuminuria 8% (20/266) vs 2% (6/265)*
PD-1 × VEGF (Ivonescimab/AK112) (29) NCT04736823 Phase II	83	Not reported	Any grade TRAE: 91.6% G3 or higher TRAE: 26.5% Decreased white blood cell count: 18.1% Neutropenia: 16.9% Decreased platelet count: 12.0% Anemia: 19.3% ALT increase: 24.1% Increased amylase: 20.5% AST increase: 20.5% Proteinuria: 21.7% Epistaxis: 16.9% Rash: 10.8% Diarrhea: 10.8%
PD-1 × VEGF (SSGJ-707) (30) NCT06361927 Phase II	83	Not reported	Any grade TRAE: 78.3% G3 or higher TRAE: 24.1% Hypercholesterolemia: 18.1% Hypertriglyceridemia: 18.1% ALT increased: 15.7% AST increased: 15.7%
PD-1 × VEGF (HB0025) (31) NCT04678908 Phase I	30	Not reported	Any grade TRAE:83.3% Grade 3 or higher TRAE: 20% Hypertriglyceridemia:23.3% Hypercholesterolemia 23.3% Anemia 16.7% ALT increase 16.7% Proteinuria 13.3%
PD-L1 × VEGF (Pumitamig/BNT327) (32)NCT05879068 Phase II	70	Not reported	G3 or higher TRAE:78.6%TRAE lead to D/C: 7.1%Two G5 TRAE (pneumonitis and immune-mediated hepatitis)Most commonly G3 or higher TRAENeutrophil count decreased: 64.3%Low white blood cell count: 35.7%
PD-L1 × VEGF (Pumitamig/BNT327) (33) NCT05844150 Phase II	50	Not reported	Any grade TRAE: 100% G3 or higher TRAE:86% Most commonly G3 or higher TRAE Neutrophil count decreased: 90.0% Anemia: 80.0% White blood cell count decreased: 76.0% Platelet count decreased: 62.0%
PD-L1 × VEGF (IMM2510) (35) NCT05972460 Phase I	33	Not reported	Any grade TRAE: 97% G3 or higher TRAE: 33.3% Infusion-related reaction: 72.7% Platelet count decreased: 39.4% Anemia: 33.3% Diarrhea: 21.2%

AE, Adverse event; AST, Aspartate aminotransferase; ALT, Alanine aminotransferase; G3, Grade 3; G5, Grade 5; Ivo, Ivonescimab; NCT, National Clinical Trial number; Pembro, Pembrolizumab; SAE, Serious adverse event; TEAE, Treatment-emergent adverse event; TRAE, Treatment-related adverse event.

*Grading according to Common Terminology Criteria for Adverse Events (CTCAE).

Additional PD-1×VEGF dual agents are also in development. SSGJ-707 showed dose-dependent activity in treatment-naïve NSCLC (NCT06361927), with ORRs ranging from 29.6% to 61.8% across different dose levels, and a tolerable safety profile (30). Similarly, HB0025 demonstrated preliminary activity in advanced solid tumors (NCT04678908), with disease control observed in 50% of evaluable patients, though response rates were modest at 9.1% (31).

On the PD-L1×VEGF bispecific side, pumitamig has shown encouraging activity in SCLC across both first-line and second-line settings. In a phase II second-line SCLC trial (NCT05879068), pumitamig plus paclitaxel achieved an ORR of 41.5% (29/70) and a DCR of 87.7% (61/70). Grade ≥ 3 adverse events occurred in 78.6% of patients, most commonly decreased neutrophil and white blood cell counts (32). In another phase II first-line extensive-stage SCLC trial (NCT05844150), pumitamig in combination with etoposide and a platinum demonstrated an ORR of 85.4% (42/48), a median PFS of 6.9 months (95% CI, 4.3–8.2), and the median OS was not yet reached. A similar toxicity profile was observed, with grade ≥ 3 adverse events occurring in 86% of patients, primarily due to decreased blood counts. Immune-related adverse events occurred in 42% of patients, with 10% being grade 3 or higher (33). Additionally, pumitamig plus chemotherapy was also evaluated in a phase II study (NCT06449209) for first or second-line SCLC. Among 38 efficacy-evaluable patients, the confirmed ORR was 76.3%, and the DCR was 100%. The median PFS was 6.8 months, while the median OS was not yet mature (34). Moreover, IMM2510 (palverafusp alfa), another PD-L1×VEGF bispecific antibody, was also being evaluated in a phase I study (NCT05972460). It demonstrated partial responses in two patients with NSCLC and exhibited a tolerable safety profile (35).

Beyond agents with clinical data discussed above, several additional PD-1/PD-L1×VEGF bispecific antibodies—such as SCTB14, JS207, RC148, AI-081, MHB039A, LM-299, B1962, AP505, and CVL006—are currently under investigation (36–39). Furthermore, building on the preliminary results observed with PD-1/PD-L1×VEGF bispecific antibodies, multiple trispecific antibodies targeting PD-1/PD-L1×VEGF×CTLA-4 (HC010, CS2009, GB268) or PD-1/PD-L1×VEGF×TGF-β (DR30206) are also in early clinical development (Appendix 1) (40–43).

### 2.2 Other potential targets

Beyond PD-1/PD-L1×VEGF, additional multi-targeting strategies are under development (Appendix 1). These include constructs that combine checkpoint inhibition with other immunomodulatory pathways, as well as TCEs and HER2-targeted bispecific antibodies. Among checkpoint-related pathways, PD-1×CTLA-4 bispecific targeting is one of the leading candidates. Cadonilimab (AK104), a PD-1×CTLA-4 bispecific, has advanced to phase III evaluation in NSCLC and is also being tested in multiple phase I/II trials across various malignancies (44). Similarly, KN046, another PD-1×CTLA-4 bispecific, is under late-phase evaluation in combination with chemotherapy or VEGF-targeted TKIs. MEDI5752, a monovalent PD-1×CTLA-4 bispecific, remains in phase I development with a large enrollment cohort (45, 46). Additionally, PD-1/PD-L1 was also studied together with other immunomodulatory pathways, including PD-1×IL-2 (IBI363), PD-1×IL-15 (SAR445877, IAP0971), PD-1×IL-18 (BPT567), PD-1×LAG-3 (Tebotelimab), PD-1×PD-L1 (IBI318), PD-1/L1×TIGIT (rilvegostomig, PM1022), PD-1×TGFβ (SHR-1701) as well as PD-1×4-1BB (GEN1046, INBRX-105) (47–57).

Beyond PD-1/PD-L1–based designs, TCEs are also in progress, including JANX008 (EGFR×CD3) for solid tumors, AMG 160 and CC-1 (PSMA×CD3) for PSMA-positive tumors, nivatrotamab (GD2×CD3) for GD2-positive malignancies, as well as RO6958688 (CEA×CD3) for solid tumors (58–62). Finally, HER2-directed bispecific antibodies, represented by ZW25 and KN026, are being evaluated in combination with other therapeutic agents (63, 64).

## 3 CAR-T therapies in lung cancer

**Figure 2:**
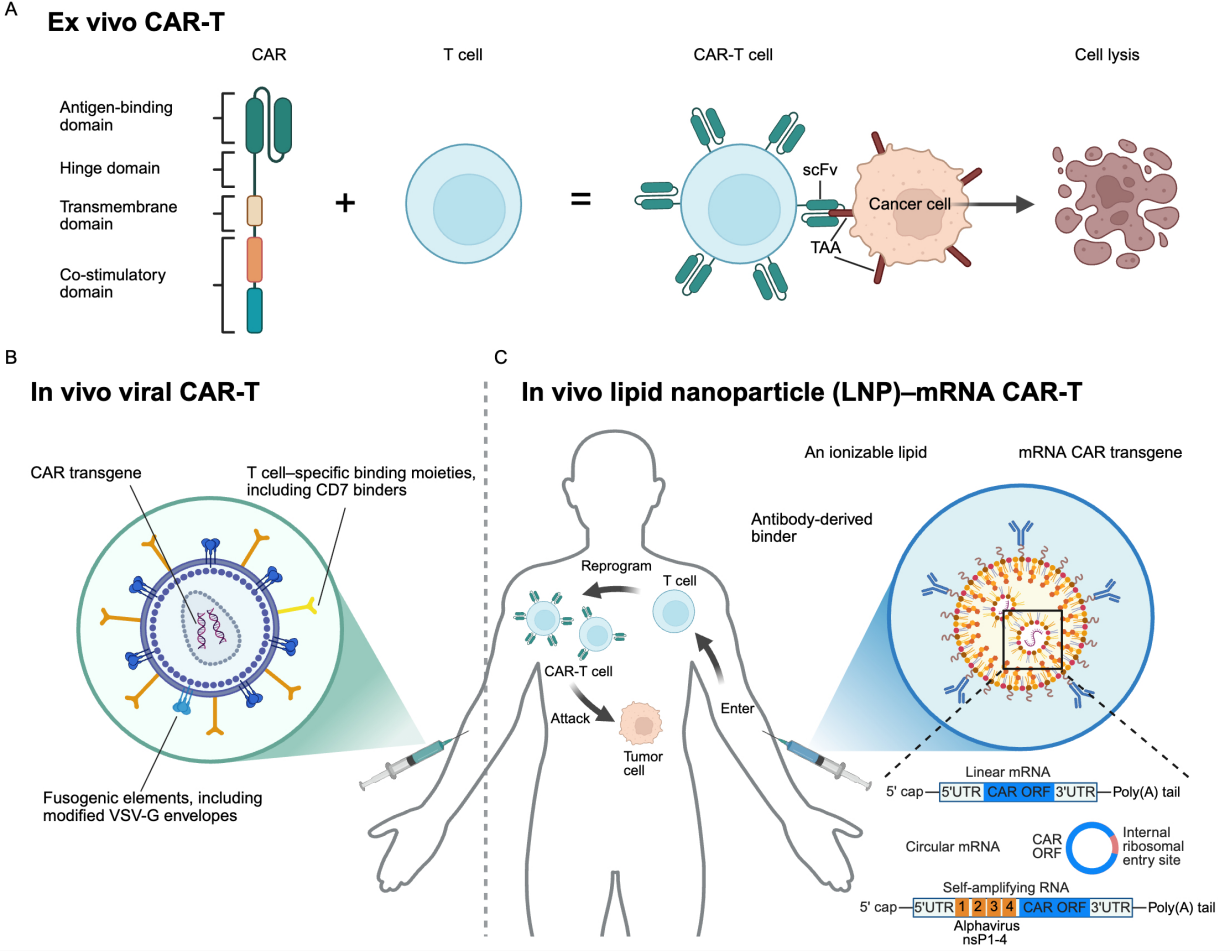
Ex vivo and *in vivo* CAR-T platforms. **(A)** Ex vivo CAR-T. CD19-targeted (tisagenlecleucel, axicabtagene ciloleucel, lisocabtagene maraleucel, brexucabtagene autoleucel, and obecabtagene), BCMA-targeted (idecabtagene vicleucel, ciltacabtagene autoleucel), and CLDN18.2-targeted (satri-cel) CAR-T therapies are autologous second-generation CAR-T products. The construction consists of an extracellular antigen-binding domain derived from a single-chain variable fragments (scFv) or nanobodies to recognize tumor-associated antigen (TAA) on cancer cells, a hinge, and a transmembrane domain that anchors the receptor and stabilizes its expression, an intracellular co-stimulatory domain—CD28 or 4-1BB—to enhance T-cell activation, proliferation, and persistence, and a CD3ζ signaling domain responsible for initiating T-cell activation and cytotoxic functions. **(B)** *In vivo* viral CAR-T (lentiviral or γ-retroviral). A retargeted strategy has been developed in which the viral envelope is engineered with T cell-specific binding moieties to enable selective uptake by T cells, along with fusogenic elements that facilitate efficient cytoplasmic delivery of the CAR transgene. The overall structural organization of these viral vectors generally parallels that of conventional ex vivo CAR-T constructs, incorporating regulatory elements to support stable and functional CAR expression. Recently, novel technologies have advanced the concept of *in vivo* CAR-T cell generation using targeted lentiviral vectors pseudotyped with either a cocal envelope protein or a CD7-targeted, modified VSV-G envelope, enabling selective delivery of the CAR transgene directly into T cells. Upon infusion, viral CAR-T vectors deliver the CAR transgene to circulating T cells in patients, which then express the receptor on their surface, reprogramming *in vivo* to recognize and eliminate tumor cells. **(C)** *In vivo* lipid nanoparticle (LNP)–mRNA CAR-T. LNPs are composed of lipid components, including an ionizable lipid that facilitates the release of mRNA-encoded CAR constructs from endolysosomes into the cytoplasm following cellular uptake. Tissue-specific targeting can be achieved by incorporating antibody-derived binders or other ligand-based moieties on the LNP surface. In LNP-based *in vivo* CAR-T, lipid nanoparticles encapsulating mRNA encoding the CAR are administered intravenously. These nanoparticles bind to circulating T cells, enter via endocytosis, and release the mRNA into the cytoplasm, where it is translated into CAR protein, transiently reprogramming the T cells to recognize and attack tumor cells. Two generations of mRNA technologies are under development: first generation, including linear mRNA, optimized for CAR expression through codon optimization, improved untranslated regions (UTRs), and chemical modifications such as N1-methylpseudouridine; Second generation, including circular mRNA (cirRNA), which lacks a 5′ cap and is designed to achieve more durable CAR expression by reducing transient expression and enhancing molecular stability; self-amplifying RNA (saRNA), which incorporates replicase-encoding sequences from alphaviruses that drive intracellular RNA replication, leading to robust and sustained transgene expression with enhanced potency and persistence in cell therapy applications. CAR, chimeric antigen receptor; ORF, open reading frame; scFv, single-chain variable fragment; TAA, tumor-associated antigen. Created with BioRender.com.

Chimeric antigen receptor (CAR) T-cell therapy has become one of the most promising anti-cancer therapies, particularly in hematologic malignancies. It utilizes engineered T cells to express a chimeric antigen receptor (CAR) that recognizes tumor antigens, triggering T-cell activation, cytokine release, and direct tumor lysis (65, 66). CAR-T products are advancing along two distinct approaches: *ex vivo* and *in vivo*. *Ex vivo* CAR-T includes autologous and allogenic designs, with the autologous design remaining the most clinically advanced. Autologous CAR-T therapy involves collecting a patient’s T cells, genetically modifying them with a CAR construct, expanding the modified cells *ex vivo*, and reinfusing them into the patient (**Figure 2A**). To date, a total of seven autologous CAR-T products have been approved by the U.S. Food and Drug Administration, including five CD19 products (tisagenlecleucel, axicabtagene ciloleucel, lisocabtagene maraleucel, brexucabtagene autoleucel, and obecabtagene). The pivotal trials of these products report high response rates and durable remissions in relapsed/refractory acute lymphoblastic leukemia (ALL) and B-cell lymphoma (67–75). Similarly, BCMA-targeted CAR-T products (idecabtagene vicleucel, ciltacabtagene autoleucel) achieved ORRs of ~70%–98% with frequent deep responses in heavily pretreated multiple myeloma (76, 77). While these CAR-T therapies provide substantial clinical benefit, toxicities such as cytokine release syndrome (CRS) and immune effector cell–associated neurotoxicity syndrome (ICANS) necessitate vigilant monitoring.

CAR-T therapies have also been used in solid tumors. Among them, satricabtagene autoleucel (satri-cel), a claudin 18.2 CAR-T, has completed its pivotal Phase II clinical trial. The randomized phase II study (NCT04581473) enrolled 156 patients with CLDN18.2-positive advanced gastric or gastro-esophageal junction cancer, including 104 treated with satri-cel and 52 who received the physician’s choice of standard therapy (nivolumab, paclitaxel, docetaxel, irinotecan, or rivoceranib). Satri-cel demonstrated superior efficacy, with a median PFS of 3.25 months (95% CI 2.86–4.53) versus 1.77 months (1.61–2.04) in the control group (HR 0.37, 95% CI 0.24–0.56; p<0.0001). The most common adverse events include decreased lymphocyte and white blood cell counts and CRS. There was no immune effector cell-associated neurotoxicity syndrome (78–80).

As with gastric cancer, clinical trials of autologous CAR-T therapy for lung cancer have explored a range of targets, including EGFR, mesothelin, MUC1, ROR1, and DLL3 (**Table 3**) (81).

**Table 3:** Safety and efficacy outcomes of autologous CAR-T therapies in lung cancer.

Product/NCT/phase	N	Diagnosis	Dose (cells/kg)	PK	Adverse Events	CRS/ICANS	Efficacy
EGFR CAR-T (82) NCT03182816 Phase I	9	Adv R/R NSCLC	2 cycles of 1 × 10^6^ or 3 × 10^6^	Detectible EGFR CAR-T in 8/9 (89%) pts	Most common AE: G1–3 fever No ³ G4 AE	No serious CRS. ICANS was not disclosed.	1 PR (lasted 13 months), 6 SD, 2 PD OS 15.63 months (95% CI, 8.82-22.03) PFS 7.13 months (95% CI:2.71-17.10)
EGFR CAR-T (83) NCT01869166 Phase I	11	Adv R/R NSCLC	0.97 × 10^7^ (Range: 0.28-2.54 × 10^7^)	Transient EGFR CAR-T detection between 11–34 days (4 pts) Sustained EGFR CAR-T detection between 7–37 weeks (7 pts)	Rash/Dry skin: 2pts (G1-2) Nausea: 1 pt (G1-2) Vomiting:1 pt (G1-2) Dyspnea: 4 pts (G1-2) Hypotension: 1 pt (G1-2) Serum amylase elevation: 1 pt (G1-2) Serum lipase elevation: 1pt (G3-4)	No CRS occurred. ICANS was not disclosed	2 PR 5 SD 4 PD
Anti-MUC1 CAR-T (84) NCT03525782 Phase I	20	NSCLC stage IIIb-IV	2.5 × 10^6^	Circulating CART cells at 20% at +4 months	Fever: 3 pts (G1-2) Chills: 2 pts (G1-2) Headache: 1 pt (G1-2) Skin rash: 1 pt (G1-2) Diarrhea: 1 pt (G1-2) Nausea/Vomiting: 1 pt (G1-2)	No ≥ G3 CRS occurred. ICANS was not disclosed	11 SD 9 PD
ROR1 CAR-T (85) NCT02706392 Phase I	18^a^	Recurrent Stage IV NSCLC or TNBC, ROR1+	3.3 × 10^5^ 1 × 10^6^ 3.3 × 10^6^ 1 × 10^7^	Not disclosed	1 NSCLC pt had respiratory failure at D17 after CAR-T infusion	CRS: 10 pts (G1-2), 4 pts (G3) ICANS: 1 pt (G1)	1 PR 16 SD 1 PD
DLL3 CAR-T (AMG 119) (86) NCT03392064 Phase I	5	R/R SCLC	3 × 10^5^ 1 × 10^6^	T_Last:_86–94 days	No ≥ G4 AE	No CRS occurred ICANS was not disclosed	1 PR 2 SD 1 PD 1 NE
DLL3 CAR-T (LB2102) (87) NCT05680922 Phase I	9^b^	R/R SCLC or LCNEC	DL1:0.3× 10^6^ DL2:1× 10^6^ DL3:2× 10^6^	Median Cmax:694.4 copies/µg genomic DNA (range, 45.6-2256.7) Median Tmax of 15 days (range, 10-29)	Anemia: 2pts(> G3), Leukopenia: 2pts(> G3) Neutropenia: 2pts(> G3),	CRS: 1pt (G1) No neurotoxicity observed	DL1: 3PD DL2: 3 SD (including 1 LCNEC pt) DL3: 1 PR, 2 SD
MSLN-targeted CAR T +/- Pembrolizumab (88) NCT02414269 Phase I	27^c^	Malignant pleural disease	1 × 10^5^ 3 × 10^5^ 1 × 10^6^ 3 × 10^6^ 6 × 10^6^ 1 × 10^7^ 3 × 10^7^ 6 × 10^7^	91% of pts with detection of CAR-T cells between 0–100 days 39% of pts with detection of CAR-T cells between 101–200 days 17% of pts with detection of CAR-T cells between 201–300 days	Constipation: 2 pts (G3) Dyspnea: 1 pt (G3) Anemia: 6 pts (G3) Lymphocyte count decreased: 16 pts (G3), 6 pts (G4) Neutrophil count decreased: 15 pts (G3), 11 pts (G4) White blood cell decreased: 13 pts (G3), 9 pts (G4) Hyperglycemia: 3 pts (G3) Hyponatremia: 2 pts (G3) Hypophosphatemia: 3 pts (G3) No grade 5 events reported	CRS: 5 pts (G1), 2 pts (G2) No neurotoxicity > G2	*23 malignant pleural mesothelioma pts receiving CAR-T* *Median OS: 17.7 months (95% CI, 13.2 to NE)* *1-year OS: 74%(95% CI, 58% to 94%)* *18 malignant pleural mesothelioma pts receiving CAR-T + Pembro* *Median OS: 23.9 months (95% CI, 14.7 to NE)* *1-year OS: 83% (95% CI, 68% to 100%)*

Adv, advanced; AE, adverse event; CAR-T, chimeric antigen receptor T-cell; CI, confidence interval; CRS, cytokine release syndrome; DL, dose level; G1–G4, grade 1 to 4 (per CTCAE v5.0); ICANS, immune effector cell–associated neurotoxicity syndrome; LCNEC, large-cell neuroendocrine carcinoma; MSLN, mesothelin; NCT, National Clinical Trial number; NE, not evaluable; NSCLC, non–small-cell lung cancer; OS, overall survival; PD, progression disease; Pembro, pembrolizumab; PFS, progression-free survival; PK, pharmacokinetics; PR, partial response; pts, patients; R/R, relapsed or refractory; SCLC, small-cell lung cancer; SD, stable disease; TNBC, triple-negative breast cancer.

^a^ Study enrollment included chronic lymphocytic leukemia (n=3) in cohort A, as well as TNBC (n=10) and NSCLC (n=8) in Cohort B. Safety and efficacy results reported as cohort B only.

^b^ Including 8 subjects with SCLC and one subject with LCNEC.

^c^ 25 patients with malignant pleural disease, 1 with metastatic lung cancer and 1 with metastatic breast cancer.

### 3.1 EGFR-targeted CAR-T in NSCLC

In a phase I study (NCT03182816) of EGFR CAR-T therapy for patients with advanced NSCLC, 9 patients received 2 cycles of CAR-T treatment at doses of 1×10^6^ or 3×10^6^ cells/kg. Clinical activity included one patient who achieved a partial response (PR) lasting 13 months, six patients with stable disease (SD), and 2 patients with progression disease (PD). The median OS was 15.6 months (95% CI, 8.8–22.0), with a median PFS of 7.1 months (95% CI, 2.7–17.1). There was no serious CRS event reported, and the most common adverse events were grade 1 or 2 fever (82). In another phase I EGFR CAR-T study (NCT01869166) in advanced NSCLC, 11 patients received doses ranging from 0.28 to 2.54 ×10^7^ cells/kg, with persistence up to 37 weeks in some patients and 2 PR, 5 SD, and 4 PD. No CRS events occurred, and the treatment was well tolerated (83).

### 3.2 Anti-MUC1 targeted CAR-T in NSCLC

Anti-MUC1 CAR-T cells were studied in patients with advanced NSCLC (NCT03525782). Twenty patients with stage IIIb–IV disease received one to four cycles of MUC1-specific CAR-T therapy at a dose 2.5×10^6^/kg. Eleven patients achieved SD, and 9 had PD. The treatment was well tolerated, with only mild adverse events, including low-grade fever, chills, headache, and rash. No grade ≥3 toxicities or CRS were observed (84).

### 3.3 ROR1-specific CAR-T in NSCLC

ROR1-directed CAR-T therapy (NCT02706392) was evaluated in 18 patients with either NSCLC (n=8) or triple-negative breast cancer (TNBC; n=10). Multiple dose levels (3.3×10^5^–1×10^7^ cells/kg) were tested, yielding 16 cases of SD, 1 PR, and 1 PD. The treatment was generally well tolerated. Four patients experienced grade 3 CRS, and no ≥ grade 2 neurotoxicity was observed. One NSCLC patient treated at the highest dose level developed dose-limiting toxicity marked by severe pulmonary inflammation and rapid CAR-T expansion, leading to fatal respiratory failure 17 days post-infusion (85).

### 3.4 DLL-3 targeted CAR-T in SCLC

Two DLL-3-directed CAR-T products were studied in the SCLC. AMG119 CAR-T was studied in five patients with relapsed/refractory SCLC (NCT03392064). It showed 1 PR, 2 SD, 1 PD, and 1 not evaluable. There were no CRS or ≥ G4 adverse events observed (86). LB2102 CAR-T was evaluated in eight patients with relapsed or refractory SCLC (NCT05680922) at dose levels ranging from 0.3 to 3.2×10^6^ cells/kg. Clinical responses included 1 PR, 4 SDs, and 3 PDs. The most common adverse events were hematologic toxicities. One grade 1 CRS event was reported, and no neurotoxicity was observed (87).

### 3.5 Mesothelin-targeted CAR-T in malignant pleural disease

Mesothelin-targeted CAR-T therapy (NCT02414269) was evaluated in 25 patients with malignant pleural mesothelioma, along with one patient with metastatic lung cancer and one with metastatic breast cancer. Treatment was administered at dose levels ranging from 1×10^5^ to 6×10^7^ cells/kg, with or without subsequent pembrolizumab. Among the 23 patients with malignant pleural mesothelioma who received cyclophosphamide plus CAR-T therapy, the median OS was 17.7 months (95% CI, 13.2–not estimable), and the 1-year OS rate was 74% (95% CI, 58–94). Eighteen of these patients subsequently received pembrolizumab, achieving a median OS of 23.9 months (95% CI, 14.7–not estimable) and a 1-year OS rate of 83% (95% CI, 68–100). Mesothelin-targeted CAR-T therapy demonstrated a favorable safety profile, with no ≥ grade 3 CRS or neurotoxicity observed (88).

Beyond the CAR-T products described above, several early-phase clinical trials in lung cancer are investigating additional targets, including CEA, PD-L1, GPC3, GD2, B7-H3, and FAP (Appendix 2) (89). These studies highlight the growing diversity of tumor-associated antigens and the effort to extend CAR-T therapy to lung cancer. Translation remains challenging due to T-cell tumor trafficking, antigen heterogeneity, an immunosuppressive tumor microenvironment, and suboptimal CAR-T persistence, which leads to early functional exhaustion and limits durable clinical responses (90–93).

To overcome the logistical and manufacturing challenges with autologous CAR-T, allogeneic and *in vivo* CAR-T platforms are being actively developed. Allogeneic CAR-T therapies, derived from healthy donors, aim to provide “off-the-shelf” availability and consistent product quality; however, they remain constrained by risks such as graft-versus-host disease and host immune rejection (94, 95).

## 4 *In vivo* CAR-T

In contrast, *in vivo* CAR-T represents a paradigm shift by enabling direct engineering of a patient’s own T cells *in situ*. This approach eliminates the need for *ex vivo* cell processing and personalized logistics and, in many cases, obviates the need for chemotherapy-based lymphodepletion, thereby improving scalability, speed, and patient access. This streamlined process allows broader applicability beyond specialized treatment centers and reduces healthcare costs (96).

To achieve *in vivo* reprogramming of T cells, two leading platforms are currently being investigated: viral vector systems and non-viral nanoparticle–based systems, each presenting distinct trade-offs in efficiency, specificity, persistence, and safety. Viral vector systems, most notably lentiviral and adeno-associated virus (AAV) vectors, have been widely explored. The pantropic vesicular stomatitis virus glycoprotein (VSV-G), commonly used to pseudotype lentiviral (LV) vectors for *ex vivo* CAR-T transduction, is less suitable for *in vivo* applications. Recently, novel technologies have advanced into clinical trials to evaluate *in vivo* CAR-T cell generation using targeted LV vectors pseudotyped with either a cocal envelope protein or a CD7-targeted, modified VSV-G envelope, enabling selective delivery of the CAR transgene to T cells (**Figure 2B**) (97–99). AAV-based approaches also allow persistent expression without integration, reducing the risk of insertional mutagenesis, although their packaging capacity and tropism constraints are limitations (100–102).

On the non-viral side, lipid nanoparticle (LNP)–based delivery enables transient CAR expression and scalable manufacturing. Still, its short-lived activity, added complexity from antibody modifications, and risk of innate immune activation remain key limitations (97, 102, 103). The LNP technology involves the rational design of nanoparticles that selectively target specific tissues. Multiple classes of lipid nanoparticles can be systematically engineered to achieve exclusive editing of extrahepatic tissues by incorporating supplemental molecules (104, 105). Examples include lung-, spleen-, and liver-targeted LNPs. This approach may extend the effective delivery window of engineered mRNA CAR-T from a few hours to several days. The evolution of mRNA technology could be classified into two generations. First-generation *in vivo* mRNA therapy often uses a modified linear mRNA structure: 5’UTR-ORF-3’UTR (106, 107). They were developed from approved SARS-CoV-2 vaccines, which enable temporary but potent protein expression, serving as a foundation for developing safer mRNA therapies with transient activity. Second-generation is exploring the advantages of more stable and potent RNA formats, such as circular mRNA (circRNA) and self-amplifying RNA (saRNA), which can lead to more durable and effective CAR-T transgene expression (**Figure 2C**) (108–111). Other advanced technologies were developed during *in vivo* mRNA development and borrowed from traditional antibody optimization, including screening a limited number of variants and using computational tools such as molecular dynamics simulations and energy functions. While these methods can improve properties such as binding affinity, they are often inefficient and limited in scope and may struggle to optimize multiple properties simultaneously due to the vast number of possible amino acid sequences. Recently, large language models, AI-powered tools, and methods have been developed, enabling rapid screening of large numbers of integrated mRNA components to develop a truly second-generation *in vivo* mRNA treatment (112–114).

With recent technological breakthroughs, the landscape of *in vivo* CAR-T therapy is rapidly evolving. Both viral and non-viral delivery platforms have now advanced into early-phase clinical evaluation (**Table 4** and Appendix 3). Many are under-evaluated without lymphodepletion. On the viral delivery side, lentivirus-based approaches are taking the lead. In a phase I trial in relapsed/refractory multiple myeloma (NCT06691685), 4 patients were treated with ESO-T01, a BCMA-directed lentivirus-based CAR-T, yielding 2 stringent complete responses and 2 PRs, although grade 3 CRS was observed in patients (115). A complete response was reported in a patient with DLBCL following treatment with a CD19-directed lentiviral *in vivo* CAR-T product (JY231) in a clinical study for relapsed or refractory B cell Lymphoma/Leukemia (116), and the major side effects included myelotoxicity and G1 CRS (117–119). Additionally, other *in vivo* CAR-T products have been studied in phase I clinical trials, including INT2104 (CD20-directed CAR-T), UB-VV111(CD19-directed CAR-T), UB-VV400 (CD22-directed CAR-T), and LVIVO-TaVEC100 (CD19/CD20 directed CAR-T in B-cell malignancies, as well as KLN-1010 (BCMA-directed CAR-T) in multiple myeloma (97, 117, 120–123). Beyond CAR-T, oncolytic viral platforms are under development, including Voyager-V1 (vesicular stomatitis virus) and MV-NIS (measles virus), which are being evaluated in multiple solid tumors, such as NSCLC, head and neck cancer, and bladder cancer, often in combination with immune checkpoint inhibitors (124–127).

**Table 4:** Overview of advanced pipelines for next-generation cell therapies.

Reference	Company	Products	Indications	Research status
Lentivirus-based delivery				
115	EsoBiotec	ESO-T01 (BCMA)	MM	Phase I ESO-T01 study in RRMM (NCT06691685) Per Xu et al., 4 RRMM pts were treated, including 2 pts with sCR and 2 pts with PR. Three pts had G3 CRS, and 1 pt had G1 CRS. One pt developed G1 ICANS.
97, 117,120,121	Interius BioTherapeutics	INT2104 (CD20), INT2106 (CD19)	CD20+ B-cell malignancies Severe autoimmune	Phase I INT2104 in B-cell malignancy w/o lymphodepletion (NCT06539338)
117,120-123	Umoja Biopharma	UB-VV111 (CD19), UB-VV400/410 (CD22), UB-VV300/310 (CD20)	NHL MM Hematology Oncology	Phase I UB-VV111 in B-cell malignancies (NCT06528301) Phase I UB-VV400 in B-cell malignancies (NCT06743503)
116-119	Genocury Biotech	JY231 (CD-19)	DLBCL Refractory autoimmune disease Systemic lupus erythematosus	Phase I JY231 in r/r B cell lymphoma/leukemia (NCT06678282) Per Chen et al., one r/r DLBCL patient achieved a complete response. The major side effects included myelotoxicity and G1 CRS. Phase I JY231 in systemic lupus erythematosus (NCT06675422) Phase I JY231 in refractory autoimmune diseases (NCT06243159, NCT07059169)
117,120	Kelonia Therapeutics	KLN-1010 (BCMA)	Multiple Myeloma	Phase I KLN-1010 in RRMM (NCT07075185)
117,121	Legend Biotech	CD19/CD20 LVIVO-TaVEC100	RR B-cell malignancies	Phase I LVIVO-TaVec100 in r/r B-cell malignancies (NCT07002112)
124-127	Vyriad*	LV169 (BCMA) Oncolytic virus platform Voyager-V1 (vesicular stomatitis virus) MV-NIS	Multiple solid tumors, including NSCLC, head and neck cancer, and bladder cancer	Phase II Voyager-V1+ cemiplimab in solid tumor (NCT04291105) Phase I/II Voyager-V1 + pembrolizumab or ipilimumab/Nivolumab in solid tumors (NCT03647163) Phase I MV-NIS in bladder cancer
Lipid nanoparticle-based delivery				
117,120,121,131	Myeloid Therapeutics	MT-303 (GPC3), MT-302 (TROP2)	HCC Metastatic epithelial tumors	Phase I MT-302 in advanced or metastatic epithelial tumor (NCT05969041) Per Lemech et al., the study confirmed successful delivery and CAR expression following systemic administration. Phase I MT-303 in GPC3-expressing cancers (NCT06478693)
117,120,132	Capstan Therapeutics	CPTX2309 (CD19)	B-cell mediated autoimmune	Phase I CPTX2309 in autoimmune disorder (healthy volunteer study; NCT06917742)
120,128,129	Strand Therapeutics	Self replicating mRNA: STX-001(IL-12), STX-003 (IL-12), Circular RNA circuit	Solid tumor including melanoma, TNBC, NSCLC and others Hematological malignancies	Phase I/II STX-001+/- pembrolizumab in advanced tumor (NCT06249048) Per Piha-Paul et al., three out of five melanoma pt achieved an objective response, including one CR, one confirmed PR, and one patient with a 100% reduction in target lesions.
117,120,130	Shenzen MagicRNA	HN2301 (CD19)	Autoimmune diseases	Phase I HN2301 in refractory systemic lupus erythematosus (NCT06801119) Per Wang et al., all five treated patients demonstrated reductions in their SLEDAI-2K scores. No grade ≥3 cytokine release syndrome (CRS) or immune effector cell–associated neurotoxicity syndrome (ICANS) events were observed.
101, 117, 120	Immorna Biotherapeutics	JCXH-213 (CD19)	B-cell non-Hodgkin lymphoma	Phase I JCXH-213 in r/r B-NHL (NCT06618313)
120, 121	Starna Therapeutics	STR-P004 (CD19)	Oncology Autoimmune diseases	Phase I STR-P004 in r/r B-NHL (NCT07003178) Phase I STR-P004 in r/r autoimmune diseases (NCT07143617)
120,133	Orna Therapeutics	ORN-101 (CD19)	B-cell driven autoimmune diseases B-cell malignancies (CD19) Sickle cell disease Beta thalassemia Infectious disease	Preclinical stage, circular mRNA
117,120	Orbital Therapeutics	OTX-201 (CD19)	Autoimmune diseases Oncology RNA vaccines	Preclinical stage, circular mRNA

BCMA, B-cell maturation antigen; B-NHL, B-cell non-Hodgkin lymphoma; CD, cluster of differentiation; CRS, cytonkine release syndrome; DLBCL, diffuse large B-cell lymphoma; HCC, hepatocellular carcinoma; ICANS, immune effector cell- associated neurotoxicity syndrome; IIT, investigator-initiated trial; MM, multiple myeloma; NHL, non-Hodgkin lymphoma; PR, partial response; NSCLC, non-small cell lung cancer; Ref, Reference; r/r, relapsed/refractory; r/r B-NHL, relapsed/refractory B-cell non-Hodgkin lymphoma; r/r DLBCL, relapsed/refractory diffuse large B-cell lymphoma; r/r MM, relapsed/refractory multiple myeloma; sCR, stringent complete response; SLEDAI-2K; Systemic Lupus Erythematosus Disease Activity Index 2000; TNBC, triple-negative breast cancer; w/o; without.

*Vyraid is also developing *in vivo* oncolytic virotherapies.

Complementing these viral approaches, non-viral LNP–based platforms are emerging as scalable and potentially safer alternatives. In an early clinical trial of heavily pretreated melanoma patients administered STX-001 (IL-12 LNP-encapsulated synthetic self-replicating mRNA), the results were encouraging, with 3 of 5 patients achieving an objective response (120, 128, 129). The phase I study of HN2301, an LNP-based CD19-directed *in vivo* CAR-T therapy for autoimmune diseases (NCT06801119), has been initiated. Five patients with systemic lupus erythematosus have received HN2301 at doses of 2 mg and 4 mg, with up to 3 administrations. At three months post-treatment, all five patients showed reductions in their Systemic Lupus Erythematosus Disease Activity Index 2000 (SLEDAI-2K) scores. No grade ≥ 3 CRS or ICANS events were reported (117, 120, 130). Additionally, the phase I study of MT-302 (TROP-2–directed CAR-T) demonstrated sufficient systemic CAR expression in patients with metastatic epithelial tumors (131). Also, the phase I study of MT-303 (GPC3-directed CAR-T) has been initiated in patients with hepatocellular carcinoma (117, 120, 121). In addition to solid malignancies, other LNP-delivered CD19 CAR-T programs (such as CPTX2309, JCXH-213, STR-P004, or OTX-201) are in the research and development stages for treating autoimmune diseases or hematologic malignancies (101, 117, 120, 121, 132, 133).

Together, the early experiences with *ex vivo* CAR-T therapy in lung cancer and the rapid advances in *in vivo* platforms highlight both the promise and the challenges of this therapeutic modality. While *ex vivo* approaches have established initial clinical feasibility, their limited scalability underscores the importance of *in vivo* strategies, which offer the potential for broader patient accessibility. Continued innovation will be essential to translate the success of CAR-T therapy in hematologic malignancies into a meaningful impact for patients with lung cancer and other solid tumors.

## 5 Conclusion

In summary, bispecific antibodies and CAR-T therapies are reshaping the immuno-oncology landscape of lung cancer through distinct yet complementary mechanisms. Bispecific antibodies have demonstrated broad clinical utility, whereas CAR-T therapies—particularly *in vivo* platforms—offer new opportunities to overcome the logistical and functional barriers of *ex vivo* manufacturing. Despite encouraging feasibility, persistent limitations in cell durability, toxicity, and tumor penetration underscore the need for continued innovation. This review did not discuss targeted therapy and antibody–drug conjugates (ADCs) in lung cancer, which other groups had reported (134–139). Integrating multispecific antibodies, *ex vivo* and *in vivo* CAR-T engineering, targeted therapy, TCRs, and ADCs will be vital to define optimal sequencing and patient selection strategies that maximize durable benefits in lung cancer.
