# Supplementary material for: Rewiring the immune response in lung cancer: current progress in bispecific antibodies, CAR-T therapy, and the rise of in vivo CAR-T platforms
Source: Front Immunol. 2026 Apr 21;17:1772428. doi: 10.3389/fimmu.2026.1772428 (PMC13139153; doi:10.3389/fimmu.2026.1772428)
Supplement: Supplementary file 1 [file Table1.docx]

Appendix 1. Therapeutic clinical trials of bispecific/multispecific antibodies in lung cancer

| Drug | Study Design | Phase | Number of Patients | NCT | | |
| --- | --- | --- | --- | --- | --- | --- |
| ***DLL3 × CD3*** | | | | | | |
| Tarlatamab | Monotherapy | III | 509 | NCT05740566 | | |
|  | Tarlatamab + PD-L1 inhibitor (Durvalumab) | III | 550 | NCT06211036 | | |
|  | Monotherapy | III | 400 | NCT06117774 | | |
|  | Monotherapy | II | 240 | NCT06745323 | | |
|  | Other Phase I/II Studies | | | NCT06598306 NCT06502977 NCT04885998 | NCT06898957 NCT05361395 | |
| BI764532 | Monotherapy | I | 282 | NCT04429087^a^ | | |
| PN328/MK6070 | Monotherapy | I/II | 232 | NCT04471727^a^ | | |
| ***Other CD3 related*** | | | | | | |
| EGFR × CD3  JANX008 | Monotherapy | I | 130 | NCT05783622^a^ | | |
| PSMA × CD3  AMG 160 | Monotherapy | I | 3 | NCT04822298^a^ | | |
| PSMA × CD3  CC-1 | Monotherapy | I/II | 3 | NCT04496674 | | |
| GD2 × CD3  Nivatrotamab | Monotherapy | I/II | 3 | NCT04750239 | | |
| CEA × CD3  RO6958688 | RO6958688+ CD20 antibody (Obinutuzumab) | I | 149 | NCT02324257^a^ | | |
|  | RO6958688+ PD-L1 (atezolizumab) | I | 228 | NCT02650713^a^ | | |
| ***EGFR x MET*** | | | | | | |
| Amivantamab | Amivantamab + EGFR TKI (Lazertinib) | III | 418 | NCT05388669 | | |
|  | Amivantamab + multiple regimens | II | 390 | NCT05498428 | | |
|  | Amivantamab + EGFR TKI (Lazertinib) or Chemo | II | 365 | NCT06667076 | | |
|  | Other Phase I/II Studies | | | NCT06083857 NCT04965090 NCT06532032 NCT05801029 NCT05488314 | NCT04077463 NCT05908734  NCT05601973  NCT06816992  NCT05299125 | |
| EMB-01 | Monotherapy | I/II | 186 | NCT03797391 | | |
|  | EMB-01 + EGFR TKI (Osimertinib) | I/II | 115 | NCT05498389 | | |
| MCLA-129 | Monotherapy | I/II | 400 | NCT04930432 | | |
| ***HER2*** | | | | | | |
| HER2 × HER2 | ZW25^c^ + Chemo | I | 279 | NCT06695845^a^ | | |
| HER2 × HER2 | KN026^c^ + KN046 (PD-1 × CTLA-4) | II | 102 | NCT04521179 ^a^ | | |
| ***HER2 × HER3*** | | | | | | |
| Zenocutuzumab | Monotherapy | II | 90 | NCT05588609^a^ | | |
| ***PD-1 × CTLA-4*** | | | | | | |
| Cadonilimab (AK104) | Monotherapy | III | 560 | NCT06617416 | | |
|  | Other Phase I/II Studies for NSCLC | | | NCT06793813 NCT06001151 NCT06424821 NCT06467500 NCT05377658 NCT06341660 NCT06331650 NCT05812534 | NCT06448910 NCT05784974 NCT06702826 NCT05816499 NCT06277674 NCT06532591 NCT06946836 | |
|  | Other Phase I/II Studies for SCLC | | | NCT05505825 NCT06406673 NCT06769971 | NCT05901584 NCT06620796 NCT06477523 | |
| KN046 | KN046 + Chemo | III | 482 | NCT04474119 | | |
|  | KN046 + Multi-target TKI (Lenvatinib) | II/III | 16 | NCT05001724 | | |
|  | KN046 + VEGF TKI (Axitinib) | II/III | 20 | NCT06020352 | | |
|  | Monotherapy | Other Phase I/II Studies | | NCT05420220 NCT04054531 | NCT03838848 | |
| MEDI5752 | Monotherapy | I | 401 | NCT03530397^a^ | | |
| ***PD-1 × IL-2*** | | | | | | |
| IBI363 | Monotherapy | I | 260 | NCT05460767 ^a^ | | |
| ***PD-1 × IL-15*** | | | | | | |
| SAR445877 | Monotherapy +/- other anticancer therapy | I/II | 291 | NCT05584670^a^ | | |
| IAP0971 | Monotherapy | I/II | 140 | NCT05396391^a^ | | |
| ***PD-1 × LAG-3*** | | | | | | |
| Tebotelimab (MGD103) | Monotherapy | I | 227 | NCT03219268 | | |
| ***PD-1 × PD-L1*** | | | | | | |
| IBI318/LY3434172 | Monotherapy | I | 103 | NCT03875157 | | |
|  | IBI318+ VEGF TKI (Lenvatinib) | II | 120 | NCT04777084 | | |
| ***PD-1/(L1) × TIGIT*** | | | | | | |
| rilvegostomig (AZD2936) | Monotherapy | III | 830 | NCT06868277 | | |
|  | rilvegostomig (AZD2936) + Chemo | III | 880 | NCT06692738 | | |
|  | rilvegostomig (AZD2936) +Chemo | III | 878 | NCT06627647 | | |
|  | rilvegostomig (AZD2936) + Trop-2 ADC( Dato-DXd) | III | 660 | NCT06564844 | | |
|  | rilvegostomig (AZD2936) + Trop-2 ADC( Dato-DXd) | III | 675 | NCT06357533 | | |
|  |  | | | NCT04995523 | NCT04612751 | |
| PM1022 | Monotherapy | I/II | 200 | NCT05867771^a^ | | |
| ***PD-1 × TGF β*** | | | | | | |
| SHR-1701 | SHR-1701 +/- Chemo | II | 107 | NCT04580498 | | |
|  | SHR-1701 + PARP inhibitor (Fluzoparib) | II | 71 | NCT04937972 | | |
| ***PD-1 × VEGF*** | | | | | | |
| Ivonescimab (AK112) | Ivonescimab (AK112)  + Chemo | III | 532 | NCT05840016 | | |
|  | Ivonescimab (AK112) + Chemo | III | 536 | NCT06928389 | | |
|  | Ivonescimab (AK112)  + Chemo | III | 1080 | NCT05899608 | | |
|  | Monotherapy | III | 780 | NCT06767514 | | |
|  | Ivonescimab (AK112) + Chemo | III | 420 | NCT06396065 | | |
|  | Other Phase I/II Studies for NSCLC | | | NCT06936644 NCT06769971 NCT06951646 | NCT04736823 NCT06196814 NCT06196814 | |
|  | Phase I/II Studies for SCLC | | | NCT06478043 NCT06820762 | NCT06620796 | |
| SSGJ-707 | SSGJ-707 + Chemo +/- PD-1/PD-L1 | II | 235 | NCT06412471 | | |
|  | Monotherapy | II | 120 | NCT06361927 | | |
| HB0025 (sotiburafusp alfa) | HB0025 (sotiburafusp alfa) + Chemo | I/II | 282 | NCT06758557^a^ | | |
|  | Monotherapy | I | 154 | NCT04678908^a^ | | |
| SCTB14 | Monotherapy | I/II | 515 | NCT06304818 | | |
| JS207 | Multiple Phase II Studies in NSCLC | | | NCT06944470 NCT06924606 | | NCT06969027 NCT06868836 |
| RC148 | RC148 +/- Chemo | I | 150 | NCT06883630 | | |
| AI-081 | Monotherapy | I/II | 204 | NCT06635785^a^ | | |
| MHB039A | Monotherapy | I/II | 196 | NCT06345482^a^ | | |
| LM-299 | Monotherapy | I/II | 108 | NCT06650566^a^ | | |
| ***PD-L1 × VEGF*** | | | | | | |
| Pumitamig (BNT327/BMS986545/PM8002) | BNT327/PMB8002 + chemo | II/III | 982 | NCT06712316 | | |
|  | BNT327/PMB8002 + chemo | III | 439 | NCT06712355 | | |
|  | BNT327/PMB8002 + chemo | III | 404 | NCT06616532 | | |
|  | BNT327/PMB8002 + B7-H3 ADC (BNT324) | I/II | 594 | NCT06892548 | | |
|  | Other Phase II Studies for NSCLC | | | NCT06841055 | NCT05756972 | |
|  | Other Phase II Studies for SCLC | | | NCT06449209 NCT05844150 | NCT05879068 | |
| Palverafusp alfa (IMM2510) | IMM2510 (Palverafusp alfa) + chemo | II | 148 | NCT06746870 | | |
|  | Phase I Studies for solid tumor | | | NCT06764836^a^ | NCT05972460^a^ | |
| B1962 | Monotherapy | II | 120 | NCT06724263^a^ | | |
| AP505 | Monotherapy | I | 35 | NCT06723964^a^ | | |
| CVL006 | Monotherapy | I | 120 | NCT06621615^a^ | | |
| SG1408 | Monotherapy | I | 216 | CTR20222623^b^ | | |
| ***PD-1 × 4-1BB*** | | | | | | |
| GEN1046 (acasunlimab) | GEN1046 (acasunlimab) +/- Pembrolizumab | II | 125 | NCT05117242 | | |
| FS222 | Monotherapy | I | 260 | NCT04740424^a^ | | |
| INBRX-105 | INBRX-105 + PD-1 (Pembrolizumab) | I/II | 160 | NCT03809624^a^ | | |
| ***PD-1 × VEFG × CTLA-4*** | | | | | | |
| HC010 | HC010 | I | 122 | NCT06307925^a^ | | |
| CS2009 | CS2009 | I | 230 | NCT06741644^a^ | | |
| GB268 | GB268 | I | 330 | NCT06934616^a^ | | |
| ***PD-L1 × VEGF × TGF β*** | | | | | | |
| DR30206 | DR30206 | I | 216 | NCT06132828^a^ | | |
| PM8003 | PM8003 | I/IIa | 78 | ChiCTR2100049391^b^ | | |
| ***CD3 related*** | | | | | | |
| PSMA × CD3 | AMG 160 | I | 3 | NCT04822298 | | |
| PSMA × CD3 | CC-1 | I/II | 3 | NCT04496674 | | |
| GD2 × CD3 | Nivatrotamab | I/II | 3 | NCT04750239 | | |
| CEA × CD3 | RO6958688+ CD20 antibody (Obinutuzumab) | I | 149 | NCT02324257^a^ | | |
| CEA × CD3 | RO6958688+ PD-L1 (atezolizumab) | I | 228 | NCT02650713^a^ | | |

Abbreviation: ADC, antibody-drug conjugate; CD3, Cluster of Differentiation 3; CEA, carcinoembryonic antigen; Chemo, chemotherapy; CTLA-4, cytotoxic T-lymphocyte–associated antigen 4; DLL3, Delta-like ligand 3; EGFR, epidermal growth factor receptor; HER2, human epidermal growth factor receptor 2; HER3, human epidermal growth factor receptor 3; NCT, National Clinical Trial number; PD-1, programmed cell death protein 1; PD-L1, programmed death-ligand 1; PSMA, prostate-specific membrane antigen; SCLC, small cell lung cancer; Sep, September; TFGβ, transforming growth factor beta; TKI, tyrosine kinase inhibitor; VEGF, vascular endothelial growth factor.

^a^ These investigational agents are listed on ClinicalTrials.gov with indications for solid tumors. They are included due to their potential relevance for patients with lung cancer.

^b^ These are clinical trials registered in China.

c Both ZW25 and KN026 are bispecific antibodies targeting two distinct HER2 epitopes (ECD2 and ECD4)
