# Supplementary material for: Rewiring the immune response in lung cancer: current progress in bispecific antibodies, CAR-T therapy, and the rise of in vivo CAR-T platforms
Source: Front Immunol. 2026 Apr 21;17:1772428. doi: 10.3389/fimmu.2026.1772428 (PMC13139153; doi:10.3389/fimmu.2026.1772428)
Supplement: Supplementary file 2 [file Table2.docx]

Appendix 2. Clinical Trials of CAR-T Therapies in Lung Cancer

| Target | Design | Study Title | Phase | N | Status | Locations | NCT |
| --- | --- | --- | --- | --- | --- | --- | --- |
| CEA | Auto CAR T | Safety and Efficacy of Chimeric Antigen Receptor T Lymphocytes for Patients With Intermediate and Advanced Tumors (AMT-116) | I/II | 39 | Unknown | China | NCT05117138 |
| CEA | Auto CAR T | A Study to Evaluate the Safety and Efficacy of A2B530, a Logic-gated CAR T, in Participants with Solid Tumors That Express CEA and Have Lost HLA-A*02 Expression (EVEREST-1) | I/II | 160 | Recruiting | US | NCT05736731 |
| CEA | Auto CAR T | A Clinical Trial Targeting CEA Chimeric Antigen Receptor T (CAR-T) for CEA Positive Advanced Malignant Solid Tumors | I | 30 | Recruiting | China | NCT06043466 |
| DLL3 | Auto CAR T | DLL3-Directed Chimeric Antigen Receptor T-cells in Subjects With Extensive Stage Small Cell Lung Cancer | I | 41 | Recruiting | US | NCT05680922 |
| DLL3 | Auto CAR T + PD-L1 | Phase I Clinical Study of αPD-L1/ DLL3 CAR-T in Patients with R/ R SCLC | I | 28 | Not Yet Recruiting | China | NCT06348797 |
| DLL3 | Auto CAR T | SNC115 Injections in Patients With Recurrent/Refractory Small Cell Lung Cancer and Lung Large Cell Neuroendocrine Carcinoma | I | 35 | Recruiting | China | NCT06384482 |
| EGFR | Allo CAR T | A Study to Evaluate the Safety and Efficacy of A2B395, an Allogeneic Logic-gated CAR T, in Participants with Solid Tumors That Express EGFR and Have Lost HLA-A*02 Expression (DENALI-1) | I/II | 240 | Not Yet Recruiting | US | NCT06682793 |
| EGFR-CXCR5 | Auto CAR T | Study of CXCR5 Modified EGFR Targeted CAR-T Cells for Advanced NSCLC | Early I | 11 | Recruiting | China | NCT05060796 |
| EGFR-CXCR5 | Auto CAR T | Study of CXCR5 Modified EGFE Chimeric Antigen Receptor Autologous T Cells in EGFR- Positive Patients With Advanced Non-small Cell Lung Cancer | I | 11 | Unknown | China | NCT04153799 |
| GD2 | Auto CAR T | Autologous CAR T-Cells Targeting the GD2 Antigen for Lung Cancer | Early I | 24 | Recruiting | US | NCT05620342 |
| GPC3 | Universal CAR T | Universal CAR-T Cells (REVO-UWD-03) for Advanced Hepatocellular Carcinoma and Lung Cancer (Wondercell-UWD3) | Early I | 60 | Recruiting | China | NCT06653023 |
| MSLN | Auto CAR T + PD-1 nano | αPD1-MSLN-CAR T Cells for the Treatment of MSLN-positive Advanced Solid Tumors | Early I | 10 | Recruiting | China | NCT04489862 |
| MSLN | Auto CAR T | A Study to Evaluate the Safety and Efficacy of A2B694, a Logic-gated CAR T, in Participants with Solid Tumors That Express MSLN and Have Lost HLA-A*02 Expression (EVEREST-2) | I/II | 230 | Recruiting | US | NCT06051695 |
| MUC1 | Auto CAR T | Phase I/II Study of Anti-Mucin1 (MUC1) CAR T Cells for Patients With MUC1+ Advanced Refractory Solid Tumor | I/II | 20 | Unknown | China | NCT02587689 |
| MUC1 | Auto CAR T | Anti-MUC1 CAR T Cells and PD-1 Knockout Engineered T Cells for NSCLC | I/II | 60 | Unknown | China | NCT03525782 |
| MUC1 | Auto CAR T | A Study of CART-TnMUC1 in Patients With TnMUC1-Positive Advanced Cancers | I | 16 | Terminated | US | NCT04025216 |
| MUC1 | Allo CAR T | P-MUC1C-ALLO1 Allogeneic CAR-T Cells in the Treatment of Subjects With Advanced or Metastatic Solid Tumors | I | 180 | Recruiting | US | NCT05239143 |
| PD-L1 | Auto CAR T + CAR TIL | Anti-PD-L1 Armored Anti-CD22 CAR-T/CAR-TILs Targeting Patients With Solid Tumors | I | 30 | Recruiting | China | NCT04556669 |
| PD-L1 | Allo CAR T | Zeushield Cytotoxic T Lymphocytes (Z-CTLs) for Relapsed or Refractory Non Small Cell Lung Cancer (NSCLC) | I | 10 | Unknown | China | NCT03060343 |
| ROR1 | Auto CAR T | Genetically Modified T-Cell Therapy in Treating Patients With Advanced ROR1+ Malignancies | I | 21 | Terminated | US | NCT02706392 |
| ROR1 | Auto CAR T | A Study to Investigate LYL797 in Adults With Solid Tumors | I | 100 | Active Not Recruiting | US | NCT05274451 |
| Mesothelin | Auto CAR T | Malignant Pleural Disease Treated With Autologous T Cells Genetically Engineered to Target the Cancer-Cell Surface Antigen Mesothelin | I/II | 113 | Active Not Recruiting | US | NCT02414269 |

Abbreviations: Auto, autologous; CAR T, chimeric antibody receptor T cells; N, number of subjects enrolled; NCT, National Clinical Trial number; PD-1, ;TIL, tumor infiltrating lymphocytes;
