# Supplementary material for: Rewiring the immune response in lung cancer: current progress in bispecific antibodies, CAR-T therapy, and the rise of in vivo CAR-T platforms
Source: Front Immunol. 2026 Apr 21;17:1772428. doi: 10.3389/fimmu.2026.1772428 (PMC13139153; doi:10.3389/fimmu.2026.1772428)
Supplement: Supplementary file 3 [file Table3.docx]

Appendix 3. Early Phase Clinical Trials of Next-generation Cell Therapies

| Company | Product | Indication | Phase | Target | N | Location | NCT |
| --- | --- | --- | --- | --- | --- | --- | --- |
| EsoBiotec | ESO-T01 | RRMM | I | BCMA | 24 | China | NCT06691685 |
| Interius Biotherapeutics | INT2104 | RR B-NHL  RR B-ALL | I | CD20 | 30 | Australia | NCT06539338 |
| Myeloid Therapeutics | MT-303 | HCC | I | GPC3 | 48 | Australia, South Korea | NCT06478693 |
| Umoja Biopharma | UB-VV111 | LBCL, CLL | I | CD19 | 106 | US, Australia | NCT06528301 |
|  | UB-VV400 | RR LBCL | I | CD22 | 70 | Unk | NCT06743503 |
| Genocury Biotech | JY231 | RR B-cell lymphoma/leukemia | I | CD19 | 20 | China | NCT06678282 |
|  |  | Active SLE | I | CD19 | 20 | China | NCT06675422 |
|  |  | Refractory autoimmune disease | I | CD19 | 20 | China | NCT06243159 |
|  |  | Refractory autoimmune disease | I | CD19 | 20 | China | NCT07059169 |
| Kelonia Therapeutics | KLN-1010 | RRMM | I | BCMA | 40 | Australia | NCT07075185 |
| Legend Biotech | LVIVO-TaVec100 | RR B-cell malignancies | I | CD19/CD20 | 30 | China | NCT07002112 |
| Vyriad | Voyager-V1+ Cemiplimab | Solid tumor | I | vesicular stomatitis virus | 87 | US, Brazil | NCT04291105 |
|  | Voyager-V1 + pembrolizumab | Solid tumor | I/II | vesicular stomatitis virus | 86 | US | NCT03647163 |
|  | MV-NIS | Bladder cancer | I | Measles | 8 | US | NCT03171493 |
| Myeloid Therapeutics | MT-302 | Adv/Met Epithelial Tumors | I | TROP2 | 48 | Australia | NCT05969041 |
|  | MT-303 | Solid tumor | I | GPC3 | 48 | Australia, South Korea | NCT06478693 |
| Capstan Therapeutics | CPTX2309 | Healthy volunteer | I | CD19 | 38 | Australia | NCT06917742 |
| Strand Therapeutics | STX-001+/- pembrolizumab | Solid tumor | I/II | IL-12 | 108 | US, Australia | NCT06249048 |
| Shenzen MagicRNA | HN2301 | Refractory SLE | NA | CD19 | 9 | China | NCT06801119 |
| Immorna Biotherapeutics | JCXH-213 | RR B-NHL | I | CD19 | 8 | China | NCT06618313 |
| Starna Therapeutics | STR-P004 | B-NHL | I | CD19 | 30 | China | NCT07003178 |
|  | STR-P004 | RR autoimmune diseases | I | CD19 | 39 | China | NCT07143617 |

Abbreviations: Adv/Met, advanced/metastatic; B-ALL, B-cell acute lymphoblastic leukemia; B-NHL, B-cell non-Hodgkin lymphoma; BCMA, B-cell maturation antigen; CLL, chronic lymphoblastic leukemia; HCC, hepatocellular carcinoma; LBCL, large B-cell lymphoma; MM, multiple myeloma; N, number of subjects to be enrolled; NA, not applicable;NCT, National Clinical Trial number; RR, relapsed/refractory; SLE, systemic lupus erythematosust; Unk, unknown;
